# Supplementary material for: Novel Bacteriophages Show Activity against Selected Australian Clinical Strains of Pseudomonas aeruginosa
Source: Microorganisms. 2022 Jan 19;10(2):210. doi: 10.3390/microorganisms10020210 (PMC8875357; doi:10.3390/microorganisms10020210)
Supplement: Supplementary file 1 [file microorganisms-10-00210-s001.zip › microorganisms-1532845-supplementary.pdf]

Supplementary Material

Novel Bacteriophages Show Activity Against Selected Australian Clinical Strains of *Pseudomonas aeruginosa*

Namonyo, S.,[1,2] Carvalho, G.,[1*] Guo, J.,[1] and Weynberg, K.D.[3*]

[1]Australian Centre for Water and Environmental Biotechnology, The University of Queensland, St. Lucia, QLD 4072, Australia
[2]Queensland Alliance for Environmental Health Services, The University of Queensland, Woolloongabba, QLD 4102, Australia
[3]Australian Centre for Ecogenomics, School of Chemistry & Molecular Biosciences, The University of Queensland, St. Lucia, QLD 4072, Australia
* Correspondence: KDW k.weynberg@uq.edu.au; GC g.carvalho@uq.edu.au

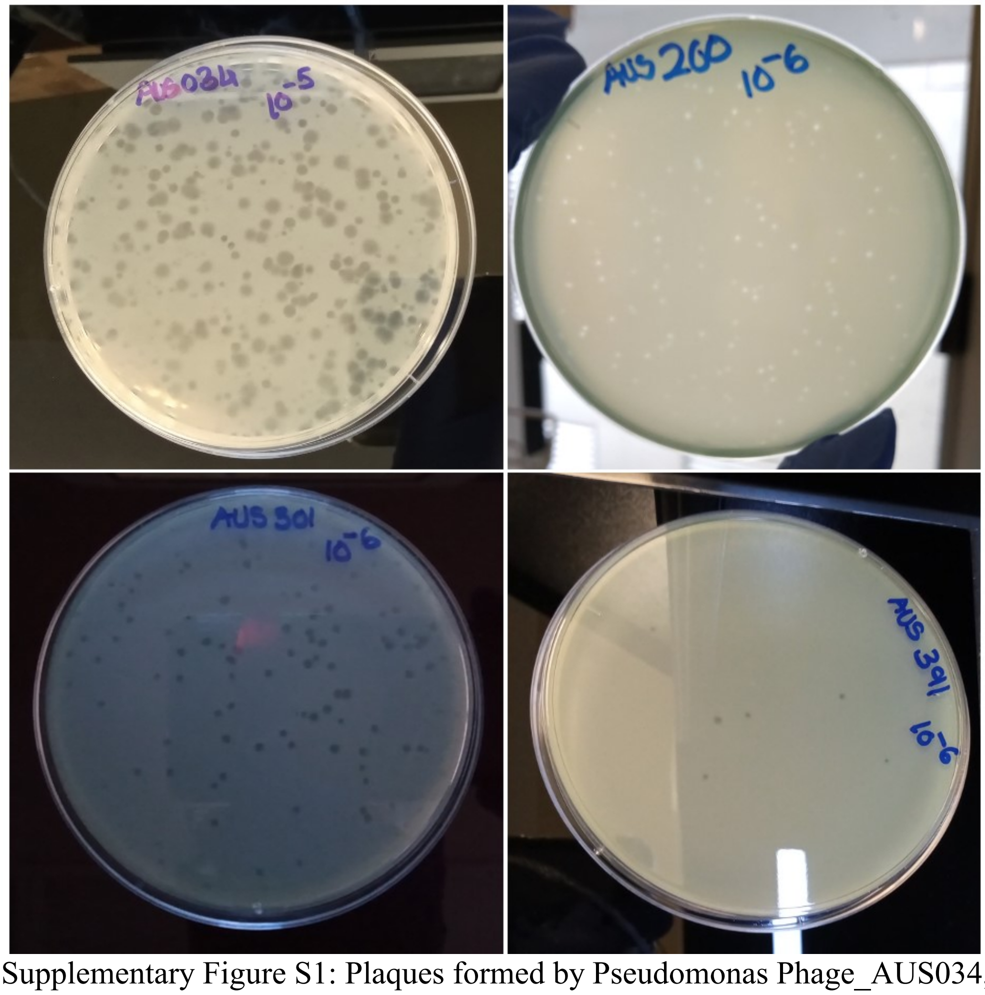

Supplementary Figure S1: Plaques formed by Pseudomonas Phage_AUS034, Pseudomonas Phage_AUS260, Pseudomonas Phage_AUS301 and Pseudomonas Phage_AUS391 respectively.

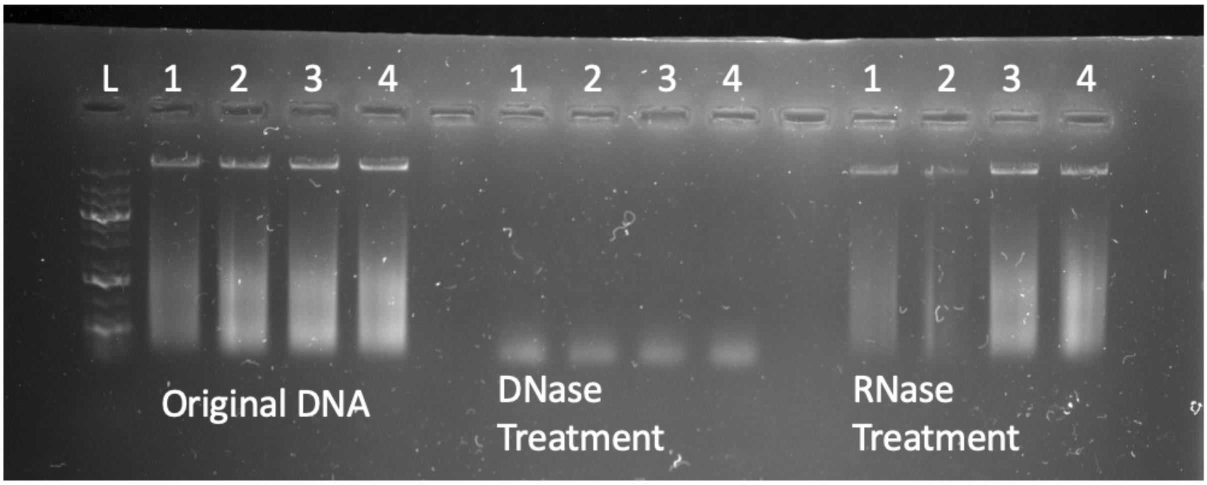

Supplementary Figure S2. Nuclease digest of the extracted phage DNA. L, 1, 2, 3 and 4 are 1Kb+ DNA Ladder, Pseudomonas Phage_AUS034, Pseudomonas Phage_AUS260, Pseudomonas Phage_AUS301 and Pseudomonas Phage_AUS391 respectively.

Supplementary Table S1: A list of the coding sequences (CDSs) and their annotations in the genome of Pseudomonas Phage_AUS034

Gene domain	Strand	Gene product	Length	e-Value	Similar to	Ontological terms (GO IDs)	InterPro GO IDs	InterPro GO Names
1630→1935	+	hypothetical protein	306	2.66E-56	Pseudomonas phage oldone	n/a	no IPS match	no IPS match
1939→2421	+	hypothetical protein	483	1.47E-111	Pseudomonas phage U47	n/a	no IPS match	no IPS match
2496→2714	+	hypothetical protein	219	9.51E-45	Pseudomonas phage vB_PaeP_C2-10_Ab22	n/a	no IPS match	no IPS match
2711→2851	+	hypothetical protein	141	4.90E-23	Pseudomonas phage PhiCHU	n/a	no IPS match	no IPS match
2980←3234	-	hypothetical protein	255	3.72E-41	Pseudomonas phage SaPL	n/a	no GO terms	no IPS match
3346→3600	+	hypothetical protein	255	5.30E-54	Pseudomonas phage SaPL	n/a	no IPS match	no IPS match
3756→3902	+	hypothetical protein	147	5.50E-26	Pseudomonas phage U47	n/a	no IPS match	no IPS match
3912→4175	+	hypothetical protein	264	2.18E-58	Pseudomonas phage SaPL	n/a	no IPS match	no IPS match
4165→4446	+	hypothetical protein	282	2.54E-61	Pseudomonas virus Pa222	n/a	no IPS match	no IPS match
4613→4864	+	hypothetical protein	252	6.80E-43	Pseudomonas phage vB_PaeP_C2-10_Ab22	C:GO:0016021	no GO terms	n/a
4861←5292	-	hypothetical protein	432	6.73E-100	Pseudomonas virus Pa222	n/a	no IPS match	no IPS match
5348→5662	+	hypothetical protein	315	1.19E-34	Pseudomonas phage SaPL	C:GO:0016021	no GO terms	n/a
5884→6846	+	hypothetical protein	963	0	Pseudomonas phage DL54	n/a	no IPS match	no IPS match
6865→7821	+	hypothetical protein	957	0	Pseudomonas phage oldone	n/a	no IPS match	no IPS match
7818→8618	+	hypothetical protein	801	0	Pseudomonas phage SaPL	n/a	no GO terms	no GO terms
8611→9180	+	hypothetical protein	570	5.57E-137	Pseudomonas virus Pa222	n/a	no IPS match	no IPS match
9156→10331	+	hypothetical protein	1176	0	Pseudomonas virus Pa222	n/a	no GO terms	no GO terms
10343→11875	+	glutamine--fructose-6-phosphate aminotransferase	1533	0	Pseudomonas phage phiPA01_302	F:GO:0004360	no GO terms	no GO terms
11885→12106	+	hypothetical protein	222	1.01E-44	Pseudomonas phage oldone	n/a	no IPS match	no IPS match
12234→13118	+	hypothetical protein	885	0	Pseudomonas virus Pa222	n/a	no GO terms	no GO terms
13118→13516	+	putative gamma-glutamyl cyclotransferase	399	2.55E-92	Pseudomonas phage SaPL	F:GO:0016740; F:GO:0061929	no GO terms	no GO terms
13516→13893	+	hypothetical protein	378	6.88E-88	Pseudomonas virus Pa222	n/a	no IPS match	no IPS match

13894→15603	+	DNA primase/ helicase	1710	0	Pseudomonas phage oldone	F:GO:0003678; F:GO:0005524; P:GO:0006260; P:GO:0032508	F:GO:0003678; F:GO:0005524; P:GO:0006260	F:DNA helicase activity; F:ATP binding; P:DNA replication
15587→16096	+	DNA polymerase III	510	2.11E-122	Pseudomonas virus Pa223	F:GO:0003676; F:GO:0004527; P:GO:0090305	F:GO:0003676	n/a
16109→16378	+	hypothetical protein	270	4.40E-50	Pseudomonas phage oldone	n/a	no GO terms	no GO terms
16410→16643	+	hypothetical protein	234	7.19E-33	Pseudomonas phage phiPA01_302	n/a	no GO terms	no GO terms
16624→16815	+	hypothetical protein	192	8.28E-38	Pseudomonas phage phiPA01_302	n/a	no IPS match	no IPS match
16805→16966	+	hypothetical protein	162	3.83E-29	Pseudomonas virus Pa222	n/a	no IPS match	no IPS match
16957→17235	+	hypothetical protein	279	4.09E-58	Pseudomonas phage U47	C:GO:0016021	no GO terms	n/a
17366→17821	+	hypothetical protein	456	4.11E-106	Pseudomonas phage DL54	n/a	no IPS match	no IPS match
17826→18023	+	hypothetical protein	198	2.28E-27	Pseudomonas phage oldone	n/a	no IPS match	no IPS match
18024→19661	+	DNA polymerase III	1638	0	Pseudomonas virus Pa222	n/a	F:GO:0003677; F:GO:0003887; P:GO:0006260; P:GO:0006261	F:DNA binding; F:DNA-directed DNA polymerase activity; P:DNA replication; P:DNA-dependent DNA replication
19731→20285	+	hypothetical protein	555	8.28E-117	Pseudomonas virus Pa222	n/a	no GO terms	no GO terms
20263→20766	+	hypothetical protein	504	3.97E-120	Pseudomonas virus Pa222	n/a	no GO terms	no GO terms
20738→21022	+	hypothetical protein	285	4.53E-61	Pseudomonas phage phiPA01_EW	n/a	no IPS match	no IPS match
21022→21906	+	Exonuclease	885	0	Pseudomonas virus Pa222	n/a	F:GO:0003677; F:GO:0003824	F:DNA binding; F:catalytic activity
21881→22867	+	putative tRNA nucleotidyltransferase/poly(A) polymerase	987	0	Pseudomonas phage U47	F:GO:0016740	no IPS match	no IPS match
22878→23060	+	hypothetical protein	183	9.07E-37	Pseudomonas virus Pa222	F:GO:0003677; F:GO:0004536; P:GO:0006259	no IPS match	no IPS match
23053→23814	+	putative metallophosphoesterase	762	0	Pseudomonas phage phiPA01_EW	n/a	no GO terms	no GO terms
23811→24029	+	hypothetical protein	219	7.92E-44	Pseudomonas phage oldone	n/a	no IPS match	no IPS match
24033→24242	+	hypothetical protein	210	7.43E-43	Pseudomonas phage PaP4	n/a	no IPS match	no IPS match
24229→24435	+	hypothetical protein	207	4.35E-22	Pseudomonas phage phiPA01_302	n/a	no GO terms	no GO terms

24580→24768	+	hypothetical protein	189	5.03E-38	Pseudomonas phage oldone	n/a	no IPS match	no IPS match
24813←25169	-	hypothetical protein	357	9.40E-80	Pseudomonas phage PaP4	n/a	no IPS match	no IPS match
25184←26071	-	putative tail fiber protein	888	0	Pseudomonas phage oldone	n/a	no IPS match	no IPS match
26083←29250	-	hypothetical protein	3168	0	Pseudomonas phage oldone	n/a	no GO terms	no GO terms
29401←30918	-	hypothetical protein	1518	0	Pseudomonas virus Pa223	n/a	no GO terms	no GO terms
30923←31303	-	particle protein	381	5.22E-55	Pseudomonas phage oldone	n/a	no GO terms	no GO terms
31303←32244	-	hypothetical protein	942	2.27E-154	Pseudomonas phage oldone	n/a	no IPS match	no IPS match
32225←32659	-	putative acetyl transferase	435	6.67E-104	Pseudomonas virus Pa222	F:GO:0016740	no IPS match	no IPS match
32656←33345	-	constituent protein	690	1.02E-142	Pseudomonas virus Pa222	n/a	no IPS match	no IPS match
33342←34883	-	hypothetical protein	1542	0	Pseudomonas phage U47	n/a	no IPS match	no IPS match
34892←35539	-	tail fibers protein	648	5.33E-138	Pseudomonas virus Pa222	n/a	no GO terms	no GO terms
35529←35777	-	hypothetical protein	249	1.37E-28	Pseudomonas phage SL4	n/a	no GO terms	no GO terms
35761←35973	-	hypothetical protein	213	1.71E-38	Pseudomonas phage Epa4	n/a	no GO terms	no GO terms
35963←36589	-	phage particle protein	627	1.14E-140	Pseudomonas phage Epa4	n/a	no GO terms	no GO terms
36593←36913	-	hypothetical protein	321	4.93E-72	Pseudomonas phage Epa4	n/a	no IPS match	no IPS match
36962←37915	-	major head protein	954	0	Pseudomonas virus Pa223	n/a	no GO terms	no GO terms
37934←38926	-	hypothetical protein	993	3.98E-152	Pseudomonas phage oldone	n/a	no GO terms	no GO terms
38926←39168	-	hypothetical protein	243	1.19E-50	Pseudomonas phage oldone	n/a	no IPS match	no IPS match
39171←41291	-	portal (connector) protein	2121	0	Pseudomonas phage LUZ24	n/a	no GO terms	no GO terms
41291←42739	-	terminase large subunit	1449	0	Pseudomonas phage phiPA01_EW	F:GO:0005524	no GO terms	n/a
42739←43137	-	T4-like lysozyme	399	1.53E-90	Pseudomonas phage phiPA01_302	n/a	no GO terms	no GO terms
43169←43627	-	terminase small subunit	459	4.18E-107	Pseudomonas phage oldone	n/a	no IPS match	no IPS match

Supplementary Table S2: A list of the coding sequences (CDSs) and their annotations in the genome of Pseudomonas Phage_AUS260

Gene Domain	Strand	Gene Product	Length	e-Value	Similar To	Ontological terms (GO IDs)	InterPro GO IDs	InterPro GO Names
1←954	-	replicative primase/helicase	954	0	Pseudomonas phage vB_PaeS-Yazdi-M	n/a	no GO terms	no GO terms
957←1328	-	hypothetical protein	372	6.52E-83	Xanthomonas phage Samson	n/a	no IPS match	no IPS match

1328→1891	+	MazG-like nucleotide pyrophosphohydrolase	564	5.46E-133	Pseudomonas phage vB_PaeS-Yazdi-M	F:GO:0016787	no GO terms	no GO terms
1967→2476	+	hypothetical protein	510	2.95E-102	Pseudomonas phage PaMx42	n/a	no IPS match	no IPS match
2629→3345	+	putative RecA ATPase	717	2.71E-175	Pseudomonas phage vB_PaeS-Yazdi-M	n/a	no GO terms	no GO terms
3484→4254	+	single-stranded DNA-binding protein	771	1.19E-92	Xanthomonas phage Samson	n/a	no GO terms	no GO terms
4423→4596	+	hypothetical protein	174	4.15E-36	Pseudomonas phage PaMx42	n/a	no IPS match	no IPS match
4765→5868	+	RecB exonuclease	1104	0	Xanthomonas phage Samson	n/a	no GO terms	no GO terms
5861→7516	+	putative helicase	1656	0	Xanthomonas phage Samson	F:GO:0003677; F:GO:0004386; F:GO:0005524	F:GO:0003677; F:GO:0005524; F:GO:0016787	F:DNA binding; F:ATP binding; F:hydrolase activity
7521→8684	+	DNA polymerase III sliding clamp	1164	0	Pseudomonas phage vB_PaeS-Yazdi-M	n/a	no GO terms	no GO terms
8696→10768	+	DNA polymerase I	2073	0	Pseudomonas phage vB_PaeS-Yazdi-M	F:GO:0000166; F:GO:0003677; F:GO:0003887; P:GO:0006260; P:GO:0039693; P:GO:0071897	F:GO:0000166; F:GO:0003676; F:GO:0003677; F:GO:0003887	F:nucleotide binding; F:nucleic acid binding; F:DNA binding; F:DNA-directed DNA polymerase activity
10765←13191	-	tail fiber protein	2427	0	Xanthomonas phage Samson	n/a	no GO terms	no GO terms
13193←14023	-	virion structural protein	831	0	Xanthomonas phage Samson	n/a	no GO terms	no GO terms
14024←15730	-	virion structural protein	1707	0	Pseudomonas phage vB_PaeS-Yazdi-M	n/a	no IPS match	no IPS match
16642←17604	-	virion structural protein	963	0	Pseudomonas phage vB_PaeS-Yazdi-M	n/a	no IPS match	no IPS match
17604←21431	-	hypothetical protein	3828	0	Pseudomonas phage vB_PaeS-Yazdi-M	n/a	n/a	no GO terms
21438←21872	-	hypothetical protein	435	3.26E-99	Xanthomonas phage Samson	n/a	no IPS match	no IPS match
21841←22143	-	tail assembly chaperone	303	1.60E-67	Pseudomonas phage vB_PaeS-Yazdi-M	n/a	no IPS match	no IPS match
22206←22634	-	tail assembly chaperone	429	2.43E-99	Pseudomonas phage vB_PaeS-Yazdi-M	n/a	no IPS match	no IPS match
22697←24238	-	major tail protein	1542	0	Pseudomonas phage vB_PaeS-Yazdi-M	n/a	no GO terms	no GO terms
24252←24707	-	tail terminator protein	456	1.02E-107	Pseudomonas phage vB_PaeS-Yazdi-M	n/a	no IPS match	no IPS match
24704←25084	-	virion structural protein	381	1.65E-86	Pseudomonas phage vB_PaeS-Yazdi-M	n/a	no IPS match	no IPS match
25088←25606	-	head-to-tail connector complex protein	519	7.23E-122	Pseudomonas phage vB_PaeS-Yazdi-M	n/a	no IPS match	no IPS match
25619←25978	-	hypothetical protein	360	4.71E-52	Pseudomonas phage vB_PaeS-Yazdi-M	n/a	no IPS match	no IPS match
26061←26468	-	hypothetical protein	408	2.22E-41	Pseudomonas phage vB_PaeS-Yazdi-M	n/a	no GO terms	no GO terms

26537←27511	-	major head protein	975	0	Pseudomonas phage PaMx42	n/a	no IPS match	no IPS match
27524←28234	-	scaffold protein	711	1.08E-158	Pseudomonas phage vB_PaeS-Yazdi-M	n/a	no GO terms	no GO terms
28272←29366	-	head morphogenesis protein	1095	0	Pseudomonas phage vB_PaeS-Yazdi-M	n/a	no GO terms	no GO terms
29369←30883	-	portal protein	1515	0	Xanthomonas phage Samson	n/a	no GO terms	no GO terms
30897←32510	-	terminase large subunit	1614	0	Xanthomonas phage Samson	n/a	no GO terms	no GO terms
32491←32979	-	putative terminase small subunit	489	4.83E-86	Pseudomonas phage PaMx42	n/a	no IPS match	no IPS match
33231←33545	-	putative Rz protein	315	3.75E-49	Pseudomonas phage PaMx42	C:GO:0016021	no GO terms	no GO terms
33545←34105	-	SAR endolysin glycosyl hydrolase	561	6.23E-119	Pseudomonas phage PaMx42	F:GO:0016787	no GO terms	no GO terms
34062←34352	-	putative holin	291	1.74E-61	Pseudomonas phage PaMx42	C:GO:0016021	no GO terms	no GO terms
34352←34720	-	hypothetical protein	369	1.39E-74	Pseudomonas phage PaMx42	C:GO:0016021	no GO terms	no GO terms
34861←35436	-	hypothetical protein	576	5.22E-106	Pseudomonas phage PaMx42	n/a	no GO terms	no GO terms
35521←35730	-	hypothetical protein	210	4.43E-26	Pseudomonas phage PaMx42	n/a	no GO terms	no GO terms
35775←36149	-	hypothetical protein	375	3.14E-84	Pseudomonas phage PaMx42	n/a	no IPS match	no IPS match
36155←36631	-	deoxycytidylate deaminase	477	4.08E-112	Xanthomonas phage Samson	F:GO:0008270; F:GO:0016787	F:GO:0003824; F:GO:0004132; P:GO:0006220; F:GO:0008270; F:GO:0016787	F:catalytic activity; F:dCMP deaminase activity; P:pyrimidine nucleotide metabolic process; F:zinc ion binding; F:hydrolase activity
36628←36861	-	hypothetical protein	234	5.95E-20	Pseudomonas phage vB_PaeS-Yazdi-M	n/a	no GO terms	no GO terms
36924←37223	-	hypothetical protein	300	3.01E-56	Pseudomonas phage vB_PaeS-Yazdi-M	C:GO:0016021	no GO terms	no GO terms
37237←37542	-	hypothetical protein	306	1.60E-51	Pseudomonas phage vB_PaeS-Yazdi-M	n/a	no GO terms	no GO terms
37599←37772	-	hypothetical protein	174	1.26E-32	Pseudomonas phage vB_PaeS-Yazdi-M	n/a	no IPS match	no IPS match
37762←37968	-	hypothetical protein	207	1.12E-42	Pseudomonas phage vB_PaeS-Yazdi-M	n/a	no IPS match	no IPS match
37968←38213	-	hypothetical protein	246	4.43E-49	Pseudomonas phage vB_PaeS-Yazdi-M	n/a	no IPS match	no IPS match
38198←38734	-	hypothetical protein	537	1.21E-118	Pseudomonas phage vB_PaeS-Yazdi-M	n/a	F:GO:0003676; F:GO:0004518	F:nucleic acid binding; F:nuclease activity
38731←38952	-	hypothetical protein	222	9.29E-45	Xanthomonas phage Samson	n/a	no IPS match	no IPS match

39010←39384	-	hypothetical protein	375	6.82E-56	Pseudomonas phage vB_PaeS-Yazdi-M	n/a	no IPS match	no IPS match
39457←39699	-	hypothetical protein	243	2.38E-51	Pseudomonas phage PaMx42	n/a	no GO terms	no GO terms
39841←40143	-	hypothetical protein	303	1.92E-68	Xanthomonas phage Samson	n/a	no IPS match	no IPS match
40492←40755	-	hypothetical protein	264	2.99E-55	Pseudomonas phage PaMx42	n/a	no IPS match	no IPS match
40772←41272	-	hypothetical protein	501	1.15E-93	Xanthomonas phage Samson	n/a	no GO terms	no GO terms
41341←41538	-	hypothetical protein	198	5.68E-39	Xanthomonas phage Samson	n/a	no IPS match	no IPS match
41535←41735	-	hypothetical protein	201	8.01E-39	Xanthomonas phage Samson	n/a	no IPS match	no IPS match

Supplementary Table S3: A list of the coding sequences (CDSs) and their annotations in the genome of Pseudomonas Phage_AUS301

Gene Domain	Strand	Gene Product	Length	e-Value	Similar To	Ontological terms (GO IDs)	InterPro GO IDs	InterPro GO Names
2←169	-	hypothetical protein	168	1.84E-32	Pseudomonas phage vB_PaeM_SCUT-S1	n/a	no GO terms	no GO terms
172←951	-	hypothetical protein	780	0	Pseudomonas phage KPP12 DNA	n/a	no GO terms	no GO terms
1038←1475	-	hypothetical protein	438	5.95E-85	Pseudomonas phage Epa20	C:GO:0016021	no GO terms	n/a
1492←2079	-	hypothetical protein	588	1.82E-144	Pseudomonas phage Epa21	n/a	no IPS match	no IPS match
2181←3113	-	hypothetical protein	933	0	Pseudomonas phage vB_PaeM_USP_3	n/a	no GO terms	no GO terms
3217←3564	-	Phage protein	348	2.44E-76	Pseudomonas phage PHW2	n/a	no GO terms	no GO terms
3813←4124	-	Phage protein	312	1.60E-67	Pseudomonas phage PASA16	n/a	no IPS match	no IPS match
4330←4653	-	Phage protein	324	4.38E-75	Pseudomonas phage vB_PaeM_E217	n/a	no GO terms	no GO terms
4685←5086	-	hypothetical protein	402	6.72E-92	Pseudomonas phage PHW2	n/a	no IPS match	no IPS match
5267→7564	+	hypothetical protein	2298	0	Pseudomonas phage Epa25	n/a	no GO terms	no GO terms
7564→8400	+	minor capsid protein	837	0	Pseudomonas phage Epa15	n/a	no GO terms	no GO terms
8419→8625	+	Phage protein	207	1.73E-28	Pseudomonas phage crassa	n/a	no GO terms	no GO terms
8622→8762	+	putative membrane protein	141	5.61E-23	Pseudomonas phage crassa	C:GO:0016021	no GO terms	n/a
9275→10708	+	putative structural protein	1434	0	Pseudomonas phage vB_PaeM_E217	n/a	no GO terms	no GO terms
10712→11347	+	putative structural protein	636	1.46E-132	Pseudomonas phage goonie	n/a	no IPS match	no IPS match
11357→12505	+	capsid and scaffold protein	1149	0	Pseudomonas phage crassa	n/a	no IPS match	no IPS match
12607→13044	+	hypothetical protein	438	1.24E-103	Pseudomonas phage vB_PaeM_USP_3	n/a	no IPS match	no IPS match

13059→13526	+	structural protein	468	4.17E-78	Pseudomonas virus PA8P1	n/a	no GO terms	no GO terms
13550→13921	+	putative structural protein	372	2.68E-83	Pseudomonas phage vB_PaeM_SMS29	n/a	no IPS match	no IPS match
13929→14480	+	structural protein	552	1.92E-133	Pseudomonas phage PASA16	n/a	no IPS match	no IPS match
14477→15058	+	phage protein	582	9.40E-126	Pseudomonas phage PASA16	n/a	no IPS match	no IPS match
15074→16588	+	putative structural protein	1515	0	Pseudomonas phage Epa7	n/a	no GO terms	no GO terms
16647→17099	+	tail fiber protein	453	3.85E-103	Pseudomonas phage Epa13	n/a	no IPS match	no IPS match
17099→17422	+	putative structural protein	324	4.06E-75	Pseudomonas phage Epa13	n/a	no IPS match	no IPS match
17419→17769	+	putative structural protein	351	5.76E-78	Pseudomonas phage PASA16	n/a	no IPS match	no IPS match
17771→18202	+	putative structural protein	432	2.91E-32	Pseudomonas phage KPP22M1 DNA	n/a	no GO terms	no GO terms
18212→18715	+	putative structural protein	504	9.16E-115	Pseudomonas phage antinowhere	n/a	no IPS match	no IPS match
18715→19254	+	putative structural protein	540	5.78E-127	Pseudomonas phage Epa13	n/a	no GO terms	no GO terms
19263→19856	+	tail fiber protein	594	3.11E-139	Pseudomonas phage antinowhere	n/a	no IPS match	no IPS match
19866→20294	+	Phage protein	429	1.63E-100	Pseudomonas phage antinowhere	n/a	no IPS match	no IPS match
20298→22874	+	putative lytic tail protein	2577	0	Pseudomonas phage S50 DNA	P:GO:0000270; F:GO:0008933; C:GO:0016020	P:GO:0000270; F:GO:0008933; C:GO:0016020	n/a
22874→23737	+	putative structural protein	864	0	Pseudomonas phage Epa7	n/a	no IPS match	no IPS match
23737→24270	+	hypothetical protein	534	4.16E-127	Pseudomonas phage Epa7	n/a	no IPS match	no IPS match
24326→24991	+	hypothetical protein	666	1.74E-134	Pseudomonas phage Epa7	n/a	no GO terms	no GO terms
25047→26300	+	putative structural protein	1254	0	Pseudomonas phage PHW2	n/a	no GO terms	no GO terms
26297→27811	+	Phage protein	1515	0	Pseudomonas phage S12-3 DNA	n/a	no GO terms	no GO terms
27789→30710	+	hypothetical protein	2922	0	Pseudomonas phage S50 DNA	n/a	no IPS match	no IPS match
30712→31140	+	putative tail fiber component	429	3.02E-99	Pseudomonas phage chunk	n/a	no IPS match	no IPS match
31140→31802	+	putative endolysin	663	9.26E-162	Pseudomonas phage PaGU11 DNA	F:GO:0004568; P:GO:0006032; P:GO:0016998	F:GO:0004568; P:GO:0006032; P:GO:0016998	F:chitinase activity; P:chitin catabolic process; P:cell wall macromolecule catabolic process
31827←32078	-	phage protein	252	1.54E-41	Pseudomonas phage Epa7	n/a	no IPS match	no IPS match
32358←33269	-	DNA ligase	912	0	Pseudomonas phage Epa7	F:GO:0003910; F:GO:0005524;	F:GO:0003910; F:GO:0005524;	F:DNA ligase (ATP) activity; F:ATP

						P:GO:0006266; P:GO:0006281; P:GO:0006310	P:GO:0006281; P:GO:0006310	binding; P:DNA repair; P:DNA recombination
33324←33878	-	putative DNA-binding protein	555	1.10E-116	Pseudomonas phage PaGU11 DNA	F:GO:0003677	no GO terms	n/a
33875←34480	-	hypothetical protein	606	2.99E-101	Pseudomonas phage vB_PaeM_LS1	C:GO:0016021	no GO terms	n/a
34534←35433	-	phage protein	900	0	Pseudomonas phage PHW2	n/a	no GO terms	no GO terms
35522←36142	-	phage protein	621	5.25E-114	Pseudomonas phage vB_PaeM_LS1	n/a	no GO terms	no GO terms
36237←37796	-	DNA helicase	1560	0	Pseudomonas phage vB_PaeM_SCUT-S1	F:GO:0004386; F:GO:0005524	F:GO:0005524; F:GO:0070615	F:ATP binding; F:nucleosome-dependent ATPase activity
37793←38203	-	putative DNA helicase	411	1.50E-94	Pseudomonas phage PaGU11 DNA	F:GO:0004386	no IPS match	no IPS match
38196←41306	-	DNA polymerase III alpha subunit	3111	0	Pseudomonas phage Epa20	F:GO:0003887; P:GO:0006260; F:GO:0008408; P:GO:0071897; P:GO:0090305	P:GO:0006260; F:GO:0008408	P:DNA replication; F:3'-5' exonuclease activity
41303←41857	-	putative DNA polymerase III epsilon subunit	555	4.31E-135	Pseudomonas phage vB_PaeM_SCUT-S1	F:GO:0003676	F:GO:0003676	no IPS match
41933←42949	-	putative polynucleotide kinase	1017	0	Pseudomonas phage vB_PaeM_LS1	F:GO:0016301; P:GO:0016310	no GO terms	no GO terms
42952←43143	-	hypothetical protein	192	1.65E-35	Pseudomonas phage PASA16	n/a	no IPS match	no IPS match
43145←44062	-	hypothetical protein	918	0	Pseudomonas phage vB_PaeM_CEB_DP1	n/a	P:GO:0006231; F:GO:0050660; F:GO:0050797	P:dTMP biosynthetic process; F:flavin adenine dinucleotide binding; F:thymidylate synthase (FAD) activity
44062←44268	-	hypothetical protein	207	4.27E-41	Pseudomonas phage vB_PaeM_PAO1_Ab27	n/a	no GO terms	no GO terms
44276←44500	-	phage protein	225	6.72E-47	Pseudomonas phage PA01	n/a	no IPS match	no IPS match
44533←44751	-	tail assembly protein	219	4.08E-46	Pseudomonas phage vB_PaeM_SMS29	n/a	no IPS match	no IPS match
44735←44953	-	phage protein	219	2.22E-32	Pseudomonas virus Pa204	n/a	no IPS match	no IPS match
44953←45183	-	phage protein	231	1.56E-49	Pseudomonas virus Pa204	n/a	no IPS match	no IPS match
45271←46272	-	hypothetical protein	1002	0	Pseudomonas virus Pa204	n/a	no GO terms	no GO terms

46376←47269	-	structural protein	894	5.22E-66	Pseudomonas virus Pa204	n/a	no GO terms	no GO terms
47427←48614	-	5' to 3' DNA helicase-like protein	1188	0	Pseudomonas phage Epa7	F:GO:0004386	no GO terms	no GO terms
48601←49023	-	hypothetical protein	423	5.49E-98	Pseudomonas phage Epa25	n/a	no IPS match	no IPS match
49120←49350	-	hypothetical protein	231	9.89E-42	Pseudomonas phage PASA16	n/a	no GO terms	no GO terms

Supplementary Table S4: A list of the coding sequences (CDSs) and their annotations in the genome of Pseudomonas Phage_AUS391

Gene Domain	Strand	Gene Product	Length	e-Value	Similar To	Ontological terms (GO IDs)	InterPro GO IDs	InterPro GO Names
2←106	-	hypothetical protein	105	4.67E-14	Pseudomonas phage PASA16	n/a	no GO terms	no GO terms
103←882	-	hypothetical protein	780	0	Pseudomonas phage KPP22M1	n/a	no GO terms	no GO terms
969←1406	-	hypothetical protein	438	6.71E-98	Pseudomonas phage KPP22M1	C:GO:0016021	no GO terms	no GO terms
1423←2010	-	hypothetical protein	588	8.81E-145	Pseudomonas phage KPP22M1	n/a	no IPS match	no IPS match
2112←3044	-	hypothetical protein	933	0	Pseudomonas phage NH-4	n/a	no GO terms	no GO terms
3148←3495	-	hypothetical protein	348	2.14E-76	Pseudomonas phage PASA16	n/a	no GO terms	no GO terms
3744←4055	-	Phage protein	312	1.60E-67	Pseudomonas phage PASA16	n/a	no IPS match	no IPS match
4061←4264	-	hypothetical protein	204	8.76E-41	Pseudomonas phage PASA16	n/a	no GO terms	no GO terms
4261←4584	-	phage protein	324	6.95E-75	n/a	n/a	no GO terms	no GO terms
4616←5017	-	hypothetical protein	402	6.72E-92	Pseudomonas phage PHW2	n/a	no IPS match	no IPS match
5198→7495	+	DUF1073 domain-containing protein	2298	0	Pseudomonas phage Epa25	n/a	no GO terms	no GO terms
7495→8331	+	minor capsid protein	837	0	Pseudomonas phage BrSP1	n/a	no GO terms	no GO terms
8350→8556	+	hypothetical protein	207	3.13E-27	Pseudomonas phage PASA16	n/a	no GO terms	no GO terms
8553→8693	+	hypothetical protein	141	6.55E-23	Pseudomonas phage PASA16	C:GO:0016021	no GO terms	no GO terms
9246→10640	+	putative structural protein	1395	0	Pseudomonas phage goonie	n/a	no GO terms	no GO terms
10644→11279	+	structural protein	636	1.39E-147	Pseudomonas phage vB_PaeM_USP_3	n/a	no IPS match	no IPS match
11289→12437	+	capsid and scaffold protein	1149	0	Pseudomonas virus Pa204	n/a	no IPS match	no IPS match
12539→12976	+	hypothetical protein	438	1.31E-103	Pseudomonas phage SL1	n/a	no IPS match	no IPS match
12991→13458	+	putative structural protein	468	2.73E-93	Pseudomonas phage willy	n/a	no GO terms	no GO terms
13455→13853	+	putative structural protein	399	3.77E-91	Pseudomonas phage crassa	n/a	no IPS match	no IPS match

13861→14412	+	structural protein	552	1.03E-132	Pseudomonas phage PASA16	n/a	no IPS match	no IPS match
14409→14990	+	phage protein	582	9.40E-126	Pseudomonas phage PASA16	n/a	no IPS match	no IPS match
15006→16520	+	putative structural protein	1515	0	Pseudomonas phage Epa7	n/a	no GO terms	no GO terms
16579→17031	+	tail fiber protein	453	3.85E-103	Pseudomonas phage Epa13	n/a	no IPS match	no IPS match
17031→17354	+	putative structural protein	324	4.06E-75	Pseudomonas phage Epa13	n/a	no IPS match	no IPS match
17351→17701	+	putative structural protein	351	5.76E-78	Pseudomonas virus Pa193	n/a	no IPS match	no IPS match
17703→18134	+	hypothetical protein	432	2.04E-33	Pseudomonas phage PASA16	n/a	no GO terms	no GO terms
18144→18647	+	putative structural protein	504	8.67E-115	Pseudomonas phage Epa25	n/a	no IPS match	no IPS match
18782→19186	+	structural protein	405	1.91E-93	Pseudomonas phage BrSP1	n/a	no IPS match	no IPS match
19195→19788	+	tail fiber protein	594	3.83E-139	Pseudomonas phage vB_PaeM_CEB_DP1	n/a	no IPS match	no IPS match
19798→20226	+	hypothetical protein	429	9.61E-101	Pseudomonas virus Pa193	n/a	no IPS match	no IPS match
20230→22806	+	putative lytic tail protein	2577	0	Pseudomonas phage sortsol	P:GO:0000270; F:GO:0008933; C:GO:0016020	no GO terms	no GO terms
22806→23669	+	putative structural protein	864	0	Pseudomonas phage Epa10	n/a	no IPS match	no IPS match
23669→24202	+	hypothetical protein	534	6.37E-128	Pseudomonas phage debbie	n/a	no IPS match	no IPS match
24258→24923	+	baseplate protein	666	5.39E-128	Pseudomonas phage Epa20	F:GO:0046872	no GO terms	no GO terms
24980→26233	+	putative baseplate protein	1254	0	Pseudomonas phage vB_PaeM_PAO1_Ab29	n/a	no GO terms	no GO terms
26230→27744	+	virion protein	1515	0	Pseudomonas phage KPP22M1 DNA	n/a	no GO terms	no GO terms
27749→30637	+	hypothetical protein	2889	0	Pseudomonas virus PA8P1	n/a	no GO terms	no GO terms
30639→31067	+	tail fiber component	429	1.02E-98	Pseudomonas virus PA8P1	n/a	no IPS match	no IPS match
31067→31729	+	lytic enzyme	663	9.46E-162	Pseudomonas phage PASA16	F:GO:0004568; P:GO:0006032; P:GO:0016998	F:GO:0004568; P:GO:0006032; P:GO:0016998	F:chitinase activity; P:chitin catabolic process; P:cell wall macromolecule catabolic process
31753←32004	-	phage protein	252	1.54E-41	Pseudomonas phage vB_PaeM_E215	n/a	no IPS match	no IPS match
32284←33195	-	DNA ligase	912	0	Pseudomonas phage vB_PaeM_CEB_DP1	F:GO:0003910; F:GO:0005524; P:GO:0006266; P:GO:0006281; P:GO:0006310	F:GO:0003910; F:GO:0005524; P:GO:0006281; P:GO:0006310	F:DNA ligase (ATP) activity; F:ATP binding; P:DNA repair; P:DNA recombination

33250←33804	-	putative DNA-binding protein	555	1.10E-116	Pseudomonas phage vB_PaeM_USP_3	F:GO:0003677	no IPS match	no IPS match
33801←34406	-	hypothetical protein	606	2.99E-101	Pseudomonas virus Pa193	C:GO:0016021	no GO terms	no GO terms
34460←35359	-	phage protein	900	0	Pseudomonas phage PASA16	n/a	no GO terms	no GO terms
35448←36068	-	hypothetical protein	621	6.74E-114	Pseudomonas phage Epa7	n/a	no GO terms	no GO terms
36163←37722	-	DNA helicase	1560	0	Pseudomonas phage DL68	F:GO:0005524	no IPS match	no IPS match
37719←38129	-	putative DNA helicase	411	1.24E-94	Pseudomonas virus Pa193	F:GO:0004386	no IPS match	no IPS match
38122←41229	-	DNA polymerase III alpha subunit	3108	0	Pseudomonas phage DL68	F:GO:0003887; P:GO:0006260; F:GO:0008408; P:GO:0071897; P:GO:0090305	P:GO:0006260; F:GO:0008408	P:DNA replication; F:3'-5' exonuclease activity
41229←41783	-	putative DNA polymerase III epsilon subunit	555	4.31E-135	Pseudomonas phage vB_PaeM_SCUT-S1	F:GO:0003676	no IPS match	no IPS match
41859←42875	-	3'-phosphatase, 5'-polynucleotide kinase	1017	0	Pseudomonas phage Epa22	F:GO:0016301; P:GO:0016310	no IPS match	no IPS match
42878←43069	-	hypothetical protein	192	1.65E-35	Pseudomonas phage PASA16	n/a	no IPS match	no IPS match
43071←43988	-	thymidylate synthase	918	0	Pseudomonas phage jett	P:GO:0006231; P:GO:0032259; F:GO:0050660; F:GO:0050797	P:GO:0006231; F:GO:0050660; F:GO:0050797	P:dTMP biosynthetic process; F:flavin adenine dinucleotide binding; F:thymidylate synthase (FAD) activity
43988←44194	-	phage protein	207	5.04E-41	Pseudomonas phage Epa10	n/a	no GO terms	no GO terms
44202←44432	-	hypothetical protein	231	1.61E-47	Pseudomonas phage Epa13	n/a	no IPS match	no IPS match
44465←44683	-	putative tail assembly protein	219	5.08E-46	Pseudomonas phage Epa13	n/a	no IPS match	no IPS match
44667←44885	-	hypothetical protein	219	4.52E-33	Pseudomonas phage Epa13	n/a	no IPS match	no IPS match
44885←45115	-	hypothetical protein	231	2.01E-49	Pseudomonas phage goonie, complete genome	n/a	no IPS match	no IPS match
45203←46204	-	hypothetical protein	1002	0	Pseudomonas phage shane	n/a	no GO terms	no GO terms
46309←47202	-	hypothetical protein	894	7.57E-65	Pseudomonas virus Pa204	n/a	no GO terms	no GO terms
47363←48550	-	DNA helicase	1188	0	Pseudomonas virus Pa193	F:GO:0004386	no GO terms	no GO terms
48537←48959	-	hypothetical protein	423	1.00E-97	Pseudomonas phage jett	n/a	no IPS match	no IPS match

49128→49913	+	putative endonuclease	786	5.85E-180	Pseudomonas phage vB_PaeM_SCUT-S1	F:GO:0004519; P:GO:0090305	no IPS match	no IPS match
49924→51153	+	hypothetical protein	1230	0	Pseudomonas phage vB_PaeM_E215	n/a	no GO terms	no GO terms
51177→51626	+	hypothetical protein	450	8.07E-106	Pseudomonas virus Pa193	n/a	no IPS match	no IPS match
51623→52699	+	putative DNA primase	1077	0	Pseudomonas phage vB_PaeM_PAO1_Ab29	n/a	no GO terms	no GO terms
52705→52890	+	putative membrane protein	186	3.35E-14	Pseudomonas phage crassa	n/a	no GO terms	no GO terms
53038→54777	+	DNA primase	1740	0	Pseudomonas virus Pa193	n/a	no GO terms	no GO terms
55749→56318	+	hypothetical protein	570	8.58E-123	Pseudomonas phage Epa20	n/a	no GO terms	no GO terms
56485←57099	-	hypothetical protein	615	8.65E-105	Pseudomonas phage shane	n/a	no GO terms	no GO terms
57291←57977	-	hypothetical protein	687	2.90E-111	Pseudomonas phage PaGU11 DNA	n/a	no GO terms	no GO terms
57989←58300	-	hypothetical protein	312	1.61E-70	Pseudomonas phage vB_PaeM_LS1	n/a	no IPS match	no IPS match
58353←58583	-	phage protein	231	7.78E-46	Pseudomonas phage PaGU11 DNA	n/a	no IPS match	no IPS match
58639←58863	-	hypothetical protein	225	7.62E-46	Pseudomonas phage goodold	n/a	no IPS match	no IPS match
58928←59254	-	hypothetical protein	327	5.29E-72	Pseudomonas phage vB_PaeM_LS1	n/a	no IPS match	no IPS match
59255←59899	-	hypothetical protein	645	1.20E-129	Pseudomonas virus Pa193	n/a	no GO terms	no GO terms
59931←60143	-	hypothetical protein	213	1.82E-45	Pseudomonas virus Pa204	n/a	no IPS match	no IPS match
60140←60340	-	hypothetical protein	201	1.11E-40	Pseudomonas virus Pa204	C:GO:0016021	no IPS match	no IPS match
60337←60552	-	hypothetical protein	216	1.33E-43	Pseudomonas virus Pa204	n/a	no IPS match	no IPS match
60549←60749	-	hypothetical protein	201	6.68E-40	Pseudomonas virus Pa204	n/a	no GO terms	no GO terms
60746←60997	-	hypothetical protein	252	5.96E-54	Pseudomonas virus Pa204	n/a	no IPS match	no IPS match
61111←61323	-	hypothetical protein	213	1.69E-41	Pseudomonas phage R12 DNA	n/a	no IPS match	no IPS match
61367←61627	-	hypothetical protein	261	2.60E-48	Pseudomonas phage PASA16	n/a	no IPS match	no IPS match
61678←61866	-	hypothetical protein	189	5.43E-39	Pseudomonas phage PASA16	n/a	no IPS match	no IPS match
61869←62555	-	hypothetical protein	687	9.43E-147	Pseudomonas phage Epa13	n/a	no GO terms	no GO terms
62608←62910	-	hypothetical protein	303	2.44E-63	Pseudomonas phage Epa13	n/a	no IPS match	no IPS match
62923←63069	-	hypothetical protein	147	5.96E-24	Pseudomonas phage Epa13	n/a	no GO terms	no GO terms
63237←63395	-	hypothetical protein	159	3.40E-31	Pseudomonas phage Epa13	n/a	no IPS match	no IPS match
63566→64948	+	hypothetical protein	1383	0	Pseudomonas phage Epa22	n/a	no GO terms	no GO terms

64985←65368	-	hypothetical protein	384	1.08E-69	Pseudomonas phage Epa22	n/a	no IPS match	no IPS match
65368←65514	-	hypothetical protein	147	1.13E-26	Pseudomonas phage PASA16	n/a	no IPS match	no IPS match
